# Testing the validity and feasibility of using a mobile phone-based method to assess the strength of implementation of family planning programs in Malawi

**DOI:** 10.1186/s12913-020-5066-1

**Published:** 2020-03-17

**Authors:** Anooj Pattnaik, Diwakar Mohan, Sam Chipokosa, Sautso Wachepa, Hans Katengeza, Amos Misomali, Melissa A. Marx

**Affiliations:** 1 Department of International Health, Johns Hopkins Bloomberg School of Public Health, 615 N. Wolfe St, E5541, Baltimore, MD 21205 USA; 2 National Statistical Office, Zomba, Malawi; 3 Reproductive Health Directorate, Ministry of Health and Population, Lilongwe, Malawi; 4 National Evaluation Platform, Lilongwe, Malawi

**Keywords:** Youth, Malawi, Implementation strength, Health worker, Family planning, Youth friendly health services, Sexual and reproductive health, Validation, Sensitivity, Specificity, Mobile phones

## Abstract

**Background:**

To effectively deliver on proposed objectives, it is vital that practitioners, policymakers, and other stakeholders are able to clearly understand how strongly their large-scale program is being implemented. This study sought to test the feasibility, cost-effectiveness, and validity of a phone-based method as an innovative and cost-efficient approach to assessing program implementation strength (through an Implementation Strength Assessment - ISA), alternative to the traditional in-person field methods.

**Methods:**

We conducted 701 mobile phone and 356 in-person interviews with facility in-Charges and two types of community health workers who provide family planning services in the Dowa and Ntcheu districts in Malawi. Responses received via the phone interview were validated through in-person review of records and inspections. Sensitivity and specificity were calculated to determine validity.

**Results:**

Most indicators at the health facility and community health worker levels were above a 70% threshold for sensitivity. However, there were fewer indicators that met this threshold for specificity. The primary reason for lower specificity was due to poor recordkeeping. Collecting data via mobile phone was found to be feasible and twice as cost-efficient as collecting the same data via in-person inspections.

**Conclusions:**

The rapid increase in mobile phone ownership and network availability in lower income countries could offer an alternative, cost-effective avenue to collect data for a better understanding of program implementation. Through rigorous assessment, this study found that using mobile phones could be a low-cost alternative to collect data on health system delivery of services, especially in places where routine data quality is poor and traditional, in-person methods are costly.

## Background

Providing family planning is shown to be one of the most effective ways of reducing maternal mortality, managing population growth, and ensuring all women have the ability to choose when to have a child [1–4]. The Sustainable Development Goals (SDGs) underline this point at a global level by including several key family planning (FP) indicators [5]. Between 1990 and 2015, modern contraceptive prevalence rates (mCPR) have increased from 54.8 to 63.3% worldwide, resulting in decreasing fertility rates, and contributing to increases in maternal and child survival around the world. However, as of 2015, mCPR is only around 40% in the Sub-Saharan African (SSA) region [6, 7].

In response, governments and non-governmental organizations (NGOs) in several SSA countries have increased their emphasis on FP programs [8, 9]. Malawi, a small, largely rural SSA country has prioritized FP over the past decade [10, 11]. In particular, the government of Malawi has emphasized targeting the youth of the country through programs, highlighted by a Youth-Friendly Health Services (YFHS) strategy. Provision of targeted SRHS to the youth through the YFHS program started in 2007, and was designed to guide programs at both health facility and community health worker levels [12].

A key challenge for countries like Malawi and other lower and middle-income countries (LMICs) that have financial, infrastructure, and human resource limitations is understanding how their FP programs are actually being implemented. A suite of tools is being developed to assist in these types of evaluations in LMICs by the Institute of International Programs (IIP) [13]. The implementation strength assessment (ISA) is one such tool that is designed to rapidly measure the quantity or dose of a program delivered to its target population and has been applied in a number of contexts [14–17]. For instance, the ISA was used in Ethiopia to show that integrated community case management of childhood illness can be implemented at scale, as nearly all health extension workers surveyed in Ethiopia were trained, supervised, and had the commodities they needed [15]. We adapted the ISA tool to assess the intensity of Malawi’s large-scale FP program implementation.

The Malawi health system delivers FP in the form of training and supervision programs, ensuring consistent FP method stocks, and activities designed to increase demand for FP at the facility and community levels. Hospitals and health centers deliver the widest range of FP commodities and demand generation activities in Malawi. At the community level, Health Surveillance Assistants (HSAs) are salaried by the Malawi government and provide counseling, condoms, oral pills, and injectables in the community. Community-Based Distribution Agents (CBDAs) are voluntary and provide counseling, condoms and oral pills in the community, as well. Each HSA and CBDA is connected to their nearest facility, where they are supposed to regularly receive supervision and commodities.

While health systems usually collect routine data on FP provision that could be used for an ISA, a common challenge is the poor quality of this data at the national and subnational levels [18–20]. Another option is collecting primary data on program implementation from each of these health system actors, but the traditional, in-person method can be labor and cost-intensive. A more cost-effective method that has been tested before is using mobile phone interviews to collect simple, quantitative data [21–23]. The increasing saturation of mobile phones among the population in SSA countries makes use of mobile phones a viable alternative to collecting data through costly field work [24]. Still, using mobile phones for data collection has its own set of challenges, such as network availability and desirability bias among respondents [23, 25].

The objective of this study is to test the validity and feasibility of collecting family planning implementation strength data at the facility and community levels using mobile phone interviews in Malawi.

## Methods

### Tool development

We developed an ISA tool for FP with extensive expert consultation on indicators and domains and as part of the National Evaluation Platform (NEP) and Real Accountability, Data Analysis for Results (RADAR), both supported by Global Affairs, Canada and technical guidance from the Institute for International Programs (IIP). RADAR’s larger objective was to develop instruments to evaluate public health programs worldwide [13, 26], while NEP aimed to build the capacity to conduct program evaluations in Malawi, Mali, Mozambique and Tanzania. The instrument is used to evaluate the implementation strength (IS) of Malawi’s FP programs across five domains of training, supervision, contraceptive method availability, demand generation activities, and accessibility. Modifications to the ISA were made to focus on FP. Previously the ISA tool had been used mostly for child health interventions. Additionally, the tool was adapted to the Malawi context. A more in-depth description of the study and tool can be found in Chipokosa et al. [27].

### Data collection

The target population was In-Charge (ICs), HSAs, and CBDAs that provide FP in two out of 28 districts in Malawi: Dowa and Ntcheu. ICs manage the health facility and thus can provide IS data for that facility. We worked in partnership with Malawi’s National Statistics Office (NSO), which recruited, trained, and oversaw data collection. Data collection took place in May 2017 after a week of training and involved two phases: phone-based and subsequently field-based data collection. Responses received via the phone interview were subsequently validated through in-person review of records and inspections of supply stocks.

### Mobile phone interviews

First, a list of ICs and their mobile phone numbers was compiled from Ministry of Health and Population district teams. Interviewers then called the ICs to elicit information about how that health facility provided FP and obtained contact information for its facility and community-level workers who provide FP. Then interviewers conducted phone interviews (VC = voice call) with all of the HSAs and CBDAs identified by the ICs. Interviews were conducted by mobile phone using tablets, and airtime was provided daily according to the number of calls they had. Supervisors conducted routine quality assurance checks of interviewer performance.

### In-person verification

Next, the interview teams conducted the in-person field verification interviews within a week of the phone interviews. All In-Charges and CBDAs were re-interviewed and a random sample of HSAs were re-interviewed. During field visits, health facility (HF) and community service provision registries, supervision records, and drug stocks were reviewed and training records were sought. Discrepancies in phone interview and in-person responses were identified during the field visit, which prompted the interviewer to ask the health worker (HW) structured qualitative questions about the reason for this discrepancy. See Table 1 for details on each IS indicator and validation methods.

**Table 1:** Description of study validation methods

Implementation Strength Indicator	HW Type	Validation method	Description
(1) Conducted mobile outreach in last year	HSA, CBDA	Monitoring or Supervisor Records	Review of register records at VC or supervisor records at HF for last year
(2) Conducted youth events in last 3 months	HSA, CBDA	Monitoring or Supervisor Records	Review of VC records or supervisor records at HF for last 3 months
(3) Have family planning Guidelines	IC, HSA, CBDA	Observation at HF or VC	Direct observation on site
(4) Provide oral contraceptive pills (OCPs)	HSA, CBDA	Observation at HF or VC	Direct observation on site
(5) OCPs available on day of interview	HSA, CBDA	Observation at HF or VC	Direct observation on site
(6) Had an OCP stockout in last 3 months	HSA, CBDA	Register Review	Review of register records at VC for previous 3 months
(7) Provide injectables	IC, HSA	Observation at HF or VC	Direct observation on site
(8) Injectables available on day of interview	IC, HSA	Observation at HF or VC	Direct observation on site
(9) Had an injectable stockout in last 3 months	IC, HSA	Register Review	Review of register records at HF or VC for previous 3 months
(10) HFs who received supervision that included FP from someone external to the HF in previous 3 reporting months	IC	Supervision records	Review of register or supervisor records at HF for previous 3 months
(11) HFs who supervise their HWs with reinforcement of YFHS practice	IC	Observation at HF	Direct observation on site
(12) HFs whose supervision checklist of HWs includes Youth FP	IC	Observation at HF	Direct observation on site
(13) HFs with Youth FP guidelines or protocols	IC	Observation at HF	Direct observation on site
(14) HFs with FP pamphlets	IC	Observation at HF	Direct observation on site
(15) HFs that provide implants	IC	Observation at HF	Direct observation on site
(16) HFs with current stocks of implants	IC	Observation at HF	Direct observation on site
(17) HFs with no stock-out in the last 3 reporting months of implants	IC	Register Review	Review of register records at HF for previous 3 months
(18) HFs have private room for FP consultations	IC	Observation at HF	Direct observation on site
(19) HFs have space designated for youth consultations & activities	IC	Observation at HF	Direct observation on site

We aimed to re-interview all the ICs and CBDAs from the two districts, and a random sample of HSAs. The sample size of 138 HSAs was based on a hypothetical indicator with a 50% prevalence at baseline that would have a sensitivity of 70% and a precision of 5%. We re-interviewed all ICs and CBDAs due to their low numbers in the two districts chosen.

### Analysis

In our analysis, the values of the in-person visits were treated as the gold standard. We calculated the proportion of health workers interviewed by phone (reported percentage) and the proportion of health workers interviewed in-person (observed percentage). Sensitivity and specificity were calculated by comparing the responses from the phone interviews to the in-person visits. The sensitivity showed the proportion of responses correctly classified by phone (e.g., reporting having stock of an item on the phone when the in-person inspection found the item was, indeed found). Specificity indicates the proportion correctly identified as NOT having the attribute (e.g., reporting by phone not having the item in stock when, upon in-person visual inspection, the item was not found in stock). An example of calculating sensitivity in this study is comparing those who self-reported (via phone interview) being trained in YFHS with those who actually have been trained in YFHS, according to the gold standard method of in-person inspection of health worker records. This analysis was done separately for the ICs, HSAs, and CBDAs. If we did not find a record of a specific indicator when checking in person, we took the conservative approach by counting that as a “No” for whether the HW conducted that activity. We established 70% sensitivity and specificity of the results of field and mobile interviews to be adequate validity. All the analyses reviewed above were conducted using R version 3.4.1 software.

### Feasibility

This study also explored the feasibility and cost-efficiency of collecting ISA data using the mobile phone by comparing the costs associated with the mobile interview phase versus the costs associated with the in-person validation phase. Key costs include the airtime used for phone interviews, equipment costs such as mobile phones and sim cards for two Malawian networks, transportation costs for in-person inspections, and other management costs such as interviewer and supervisor per diems. Ultimately, we aimed to compare cost-per-interview using mobile phones versus in-person inspections. We also analyzed feasibility at the system level, which includes network availability and the reported percentage.

### Ethical consideration

The Johns Hopkins Bloomberg School of Public Health Institutional Review Board and the Malawi National Health Science Research Committee approved the ISA validation study in April 2017. Verbal informed consent was obtained from all study participants.

## Results

We reached all 59 (100%) In-Charges that manage the hospitals and health centers in the districts of Dowa and Ntcheu both on the phone and in-person. There were 7 facilities that stated that they do not provide FP. Phone interviews were conducted with 529 (96%) HSAs and 113 (97%) CBDAs. In-person interviews were conducted with 109 (94%) CBDAs and the random sample of the 529 total HSAs (188 HSAs).

Table 2 provides an overview of the reported and observed percentages, as well as the sensitivity and specificity, for the health facility IS indicators. We were not able to validate the training indicators because we could not find consistent, organized training records for health workers at the health worker, facility, or district levels. Several indicators have lower totals for the reported and observed percentages because they were based on whether the respondent said yes to a previous question. Sensitivity for the supervision indicators was above the threshold for external supervision (80%), YFHS supervision (100%), and supervision checklist that includes youth topics (75%). However, specificity for each of these indicators was below the threshold (50, 66, and 31% respectively). Indicators pertaining to FP supplies showed the same pattern.

**Table 2:** Implementation strength indicators reported by the In-Charges versus observed by the interviewers with sensitivity and specificity of phone interview method

Implementation Strength Indicator	Reported percentage (n/N)	Observed Percentage (n/N)	Sensitivity (%)	Specificity (%)
HFs who received FP supervision from someone external in previous 3 months	73 (38/52)	77 (40/52)	80	50
HFs who supervise their HWs with reinforcement of YFHS practice	49 (18/37)	22 (8/37)	100	66
HFs whose supervision checklist of HWs includes youth FP	69 (25/36)	89 (32/36)	75	31
HFs with FP guidelines	94 (50/53)	85 (45/53)	96	13
HFs with youth FP guidelines	57 (31/54)	45 (24/53)	73	52
HFs with FP job aids	86 (44/51)	61 (31/51)	94	25
HFs with FP pamphlets	75 (39/52)	73 (38/52)	89	50
HFs that provide				
Injectables	96 (51/53)	96 (51/53)	100	100
Implants	88 (45/51)	96 (49/51)	95	100
HFs with current stocks of				
Injectables	96 (49/51)	96 (49/51)	100	100
Implants	74 (39/53)	77 (41/53)	100	67
HFs with no stock-out in the previous 3 reporting months of				
Injectables	88 (45/51)	96 (49/51)	100	92
Implants	87 (34/39)	92 (36/39)	67	92
HFs that have a private room for FP consultations	91 (48/53)	89 (47/53)	94	33
HFs have a space designated for youth consultations and activities	13 (7/53)	21 (11/53)	45	95

There was higher sensitivity and specificity for the FP method indicators. The providing injectables indicators demonstrated sensitivity and specificity of 100%, while providing implants was 95 and 100% respectively. Sensitivity and specificity was 100% for the indicator of whether injectables were available on the day of the interview. The indicator of whether implants were available on the day of the interview had a sensitivity of 100%, though just below the threshold for specificity (67%). The indicator for whether the facility experienced any stockouts of injectables in the previous 3 months was also 100% for sensitivity and 92% for specificity. Sensitivity for stockouts of implants was just below the threshold (67%), but 92% for specificity.

Table 3 provides an overview of the reported and observed percentages, as well as the sensitivity and specificity, of the IS indicators for HSAs and CBDAs. The indicator for mobile outreach showed high sensitivity (83%) and low specificity (39%) among HSAs, and the opposite pattern among CBDAs (47 and 76% respectively). The demand generation indicator of having recently conducted youth events demonstrated high sensitivity and low specificity among HSAs (80 and 48%) and CBDAs (91 and 24%). The indicators for FP supplies had high sensitivity but very low specificity for both HSAs and CBDAs. For instance, the sensitivity for FP guidelines was 98% among HSAs and 99% among CBDAs, while specificity for this indicator was 8% for HSAs and 0% for CBDAs.

**Table 3:** Implementation strength indicators reported by HSAs and CBDAs versus observed by the interviewers with sensitivity and specificity of phone interview method

Implementation Strength Indicator	Reported Percentage (n/N)	Observed Percentage (n/N)	Sensitivity (%)	Specificity (%)
Health Surveillance Agents (HSAs)				
Conducted mobile outreach in last year	71 (134/188)	47 (88/188)	83	39
Conducted youth events in last 3 months	56 (105/188)	13 (25/188)	80	48
Have family planning Guidelines	96 (181/188)	65 (123/188)	98	8
Have family planning job aids	79 (149/188)	70 (131/188)	89	44
rovide oral contraceptive pills (OCPs)	55 (104/188)	63 (118/188)	70	70
OCPs available on day of interview	87 (72/83)	80 (66/83)	92	35
Had a OCP stockout in last 3 months	28 (23/83)	19 (16/83)	69	82
Provide injectables	69 (129/188)	83 (156/188)	77	72
Injectables available on day of interview	93 (111/120)	89 (107/120)	98	54
Had an injectable stockout in last 3 months	23 (27/120)	19 (23/120)	74	90
Community-Based Distribution Agents (CBDAs)				
Conducted mobile outreach in last year	30 (33/109)	28 (30/109)	47	76
Conducted youth events in last 3 months	83 (91/109)	50 (55/109)	91	24
Have family planning Guidelines	99 (108/109)	85 (93/109)	99	0
Have family planning job aids	83 (90/109)	87 (95/109)	84	29
Provide oral contraceptive pills (OCPs)	71 (77/109)	81 (88/109)	82	76
OCPs available on day of interview	92 (66/72)	86 (62/72)	95	30
Had a OCP stockout in last 3 months	47 (34/72)	39 (28/72)	70	66

The indicator for providing oral contraceptive pills (OCPs) was above the threshold for sensitivity among HSAs (70%) and CBDAs (82%), and specificity among HSAs and CBDAs as well. The indicator for availability of OCPs on the day of interview demonstrated high sensitivity for both HSAs (92%) and CBDAs (95%), but low specificity (35% for HSAs and 30% for CBDAs). The indicator for OCP stockouts hovered around the threshold, with sensitivity at 69% for HSAs and 70% for CBDAs, and specificity at 82% for HSAs and 66% for CBDAs. Sensitivity and specificity was above the threshold for HSAs providing injectables (77 and 72%), higher sensitivity (98%) and lower specificity (54%) for availability on day of interview, and above the threshold for both sensitivity (74%) and specificity (90%) for the recent injectable stockout indicator. Overall, HSAs and CBDA reported similarly across both data collection methods, except for the mobile outreach and OCP stockout indicators.

During the qualitative questioning following the in-person inspection, many respondents admitted that they did not clearly understand what certain questions were asking on the phone. For instance, respondents were often unsure of the exact definition of youth events, or the difference between guidelines and job aids. This confusion occurred more often at the HSA and CBDA levels, where training and education is lower. Still, the most frequent reason that there was a discrepancy between phone and in-person interviews among all HW types was because of a lack of records for verification. Many health workers either had no way of tracking certain activities, such as demand-generation activities, or simply did not consistently mark these activities in their tracking sheets.

### Feasibility

We found that the cost per mobile interview was $10.56 (or 7655 Kwacha), while the cost per in-person interview was $25.48 (18,473 Kwacha). One of the largest drivers of cost in the mobile interview phase stemmed from the airtime used. The biggest driver of cost in the in-person phase was transportation to the inspection sites. Management costs comprised a substantial chunk of the costs in both mobile phone and in-person interviews, but didn’t differ substantially between them. At the systems level, we reached all 59 In-Charges, 96% (529) HSAs on the phone and 97% (113) CBDAs.

## Discussion

Our study showed that nearly all health workers that provide FP in several districts in Malawi could be interviewed on the phone. The majority of ISA indicators at the health facility, HSA, and CBDA levels in Malawi were above the 70% threshold for sensitivity. However, there were fewer indicators that met this threshold level for specificity. There were also certain indicators, such as for FP guidelines, where specificity was so low because so few respondents answered no. Aside from this, the major reason for lower specificity for the remaining indicators was due to poor recordkeeping.

The indicators for FP commodities had much higher sensitivity and specificity. This is largely because all ICs and HWs have an FP register that they have been trained to fill out and submit on a regular basis. When we conducted the in-person inspection, we quickly realized that these commodity indicators were the only ones that HWs consistently recorded. There were inconsistent records for supervision, demand generation activities, and mobile outreach. Even so, the IC kept more records of these indicators than the HSAs and CBDAs. Similar studies, such as Hazel et al., demonstrated higher sensitivity and specificity largely because they evaluated a very specific program that had been recently implemented with clear, measurable components [14]. The ISA used for this study was much broader, as it aimed to assess multiple FP programs implementing a wider set of FP practices. Consequently, the target population often kept incomplete or inconsistent records of their implementation; making it a poor choice of gold standard.

Future studies should carefully understand what records different levels of HWs keep and whether certain indicators can be validated or choose another gold standard option. Moreover, this finding also demonstrates that quality and consistency of recordkeeping in Malawi for these indicators needs to be improved for better tracking and understanding of implementation. Perhaps multiple methods of verification could be used (such as following up with community members on whether the HSA conducted a youth event in the last 3 months) rather than just the single method of checking written records.

Another reason that likely contributed to lower specificity for certain indicators was the respondents’ confusion with technical terms. During the qualitative questioning following the in-person inspection, many respondents admitted that they did not clearly understand what certain questions were asking about. We recommend that future studies conduct a pre-test or qualitative survey to understand what the confusing terms may be at the different HW levels in that context and revise the survey questions accordingly. Also, future program managers should train data collectors to clarify potentially confusing terms when asking questions of the respondent. If certain indicators are prone to confusion through the phone call method, they may be more suitable to in-person visits. To offset the cost and capacity implications, perhaps these indicators could be collected less frequently. There could be utility in hybrid data collection methods where certain indicators are collected more routinely via phone, whereas others are collected less frequently via in-person visits but a wider range of data is collected in person.

Another threat to validity is the potential desirability bias among respondents during phone interviews [28, 29]. In other words, respondents may be more likely to give answers that they believe data collectors want to hear rather than giving truthful answers that they would ordinarily give if the data collector was in front of them. We did not think social desirability would affect the more objective ISA (structural quality) measures, like they might for other more subjective types of measures. Nevertheless, we cannot confirm that in-person interviews would have been more accurate in our study because of the lack of consistent recordkeeping by all three types of HWs. An alternative that would be more accurate is to directly observe health workers over time to record ISA indicators, which would be prohibitively time and resource-intensive. While several studies have shown that using mobile phone interviews for data collection provided accurate results at cheaper costs, further research should be conducted to explore whether respondents are more likely to give socially desirable responses over the phone, even when questions are about fairly objective attributes measuring structural quality [23, 25].

Higher sensitivity and lower specificity means that this method could lead to more false positives, hence a potential overestimation of implementation strength. This finding could have implications for program managers and decisionmakers, as they might assume that certain areas do not need stronger implementation. Still, the preference is for higher sensitivity because in a resource-limited country like Malawi, knowing which areas suffer very poor IS allows for prioritization of attention. Especially in a context where records are poorly maintained, collecting data via mobile phones at least gives decisionmakers a closer approximation of how programs are being implemented.

On the feasibility end, collecting data via in-person interviews was found to be over double the cost per interview versus collecting the same data using mobile phone interviews. One major advantage of the mobile phone interview method is that it can be conducted from a central location, it saves on transportation costs and supervision is more consistent. A sizeable portion of the cost associated with the mobile interview method stem from purchasing equipment such as the mobile phones, sim cards, and headsets. However, these are a one-time purchase so any future data collection exercises using this call center approach will not have this cost and be even more cost-efficient. Furthermore, network availability and mobile phone saturation will only continue to improve as time goes by. Note that the costs analyzed and reported are specific to the Malawi context, though we do not anticipate significant differences in the cost comparison between mobile phone and in-person interviews in other contexts. In fact, Malawi is a relatively small, dense country and we therefore would expect transport costs to increase in other, larger contexts. This study provides an example of how a low-income country with significant resource constraints still has sufficient capacity, network, and mobile phone saturation (specifically among health workers) to conduct ISA interviews using this method. The significant cost savings from the mobile phone method adds to the debate about the tradeoff between validity and feasibility for conducting ISA interviews. This study suggests that it is much more cost-effective to use the mobile phone method for the indicators that demonstrated validity above the 70% threshold; for instance, tracking commodities. In contexts with poor quality routine data, the cost savings from this call center approach can prompt more rapid primary data collection and better inform policymakers and program managers of how their programs are being implemented.

### Limitations

The first limitation is that the districts of Dowa and Ntcheu that were chosen purposefully for logistical reasons. Although they are in the same Central region, fairly similar in terms of demographic characteristics, level of urbanization and supply-side performance, there still could be potential confounders that could contribute to differences in validity and feasibility between the two districts. Local government staff members were consulted and the recent DHS was reviewed to understand any key differences between the districts.

There could also be interviewer bias, where some interviewers ask or clarify survey questions more clearly. While the supervisors of each team were trained to closely monitor this, the relative simplicity of this quantitative survey also argues against such bias having a substantial effect. Still, future data collection supervisors should standardize data collector’s responses during training and oversee interviews in order to correct deviations throughout data collection to improve standardization and reduce the differences in data collection.

Another potential limitation of this study is that interview teams needed to obtain contact information for the health workers from In-Charges prior to actually conducting the interviews. These In-Charges could inform the providers that the interview team will be calling them and this could affect their responses. Similarly, we informed the health workers during the consent process prior to the interview that an inspection visit will occur at their health facility or village clinic to check their responses. Some health workers could have made changes to their records or supply stocks to make it appear that they have reported accurately. Health workers being interviewed may not trust interviewers when they say that that their responses will not be reported back to their supervisor. Despite assurances made during the consent process to the contrary, they might be worried that their responses may adversely affect their employment. While we may think these concerns may be more acute for phone-based interviews, and indeed, other studies have shown that response rates are lower for mobile phone data collection versus in-person [30], we did not experience lower response rates on phone vs. in person. We think our response rate was high for a number of reasons, including that the IC informed her HWs that we would call, the short and simple nature of our survey, and the fact that we interviewed health workers, who are more likely to have a phone than the general population who may or may not be employed.

Another limitation covered earlier is using the records of health workers as the gold standard to test for validity. These records themselves are prone to error and were often incomplete. However, this was the best choice available for validating the mobile phone interview method. Future studies could explore other data collection methods such as computer-assisted telephone interviews (CATI), interactive-voice response (IVR), and short message service (SMS).

## Conclusions

The rapid increase in mobile phone ownership and network availability in lower income countries could offer an alternative, cost-effective avenue to collect data for a better understanding of program implementation. However, there is still uncertainty about the validity and feasibility of remote data collection in lower income countries, especially among health workers [14, 31]. This study tests whether using this m-Health method can produce valid IS data and can this be a feasible alternative to traditional data collection methods. While there are challenges around validation methods, we found that using mobile phones could be a low-cost alternative to collect data on health system delivery of services, especially in places where routine data quality is poor and traditional, in-person methods are costly. This could give policymakers and program managers an often updated data source from which they can assess implementation progress and inform data-driven decision-making at the most granular levels.

## Data Availability

The datasets used and/or analyzed during the current study are available from the corresponding author on reasonable request.

## Abbreviations

CBDA: Community-Based Distribution Agent
FP: Family planning
HF: Health facility
HSA: Health Surveillance Assistant
HW: Health worker
IC: In-Charge
IIP: Institute for International Programs
ISA: Implementation strength assessment
LMICs: Lower and middle-income countries
mCPR: Modern contraceptive prevalence rate
NEP: National Evaluation Platform
NGO: Non-Governmental Organization
NSO: National Statistics Office
OCPs: Oral contraceptive pills
RADAR: Real Accountability, Data Analysis for Results
SDGs: Sustainable Development Goals
SSA: Sub-Saharan Africa
VC: Voice call
YFHS: Youth-Friendly Health Services

## References

[1] Singh S, Darroch JE (2014). Adding it up: costs and benefits of contraceptive services. Estimates for 2014.

[2] Ahmed S, Li Q, Liu L, Tsui AO (2012). Maternal deaths averted by contraceptive use: an analysis of 172 countries. Lancet.

[3] Tsui AO, McDonald-Mosley R, Burke AE (2010). Family planning and the burden of unintended pregnancies. Epidemiol Rev.

[4] Loaiza E, Blake S (2010). How universal is access to reproductive health? A review of the evidence.

[5] Starbird E, Norton M, Marcus R (2016). Investing in family planning: key to achieving the sustainable development goals. Glob Health Sci Pract.

[6] Cleland J, Conde-Agudelo A, Peterson H, Ross J, Tsui A (2012). Contraception and health. Lancet.

[7] Alkema L, Kantorova V, Menozzi C, Biddlecom A (2013). National, regional, and global rates and trends in contraceptive prevalence and unmet need for family planning between 1990 and 2015: a systematic and comprehensive analysis. Lancet.

[8] Bongaarts J, Hardee K (2017). The role of public-sector family planning programs in meeting the demand for contraception in sub-Saharan Africa.

[9] Kuang B, Brodsky I (2016). Global trends in family planning programs, 1999–2014. Int Perspect Sex Reprod Health.

[10] Government of Malawi (2015). Malawi costed implementation plan for family planning, 2016–2020.

[11] Malawi Health Sector Strategic Plan 2011–2016 (2011). Moving towards equity and quality.

[12] Government of Malawi (2015). Youth-friendly health services strategy, 2016–2020.

[13] Real Accountability: Data Analysis for Results (RADAR). 2018. Retrieved from https://www.jhsph.edu/research/centers-and-institutes/institute-for-international-programs/current-projects/RADAR/index.html. Accessed 23 June 2018.

[14] Hazel E, Amouzou A, Park L, Banda B, Chimuna T, Guenther T (2015). Real-time assessments of the strength of program implementation for community case management of childhood illness: validation of a mobile phone-based method in Malawi. Am J Trop Med Hyg.

[15] Miller NP, Amouzou A, Tafesse M, Hazel E, Legesse H, Degefie T (2014). Integrated community case management of childhood illness in Ethiopia: implementation strength and quality of care. Am J Trop Med Hyg.

[16] Bryce J, Victora CG, Boerma T, Peters DH, Black RE (2011). Evaluating the scale-up for maternal and child survival: a common framework. Int Health.

[17] Bruce J (1990). Fundamental elements of the quality of care: a simple framework. Stud Fam Plan.

[18] O'Hagan R, Marx MA, Finnegan KE, Naphini P, Ng'ambi K, Laija K, Wilson E, Park L, Wachepa S, Smith J, Gombwa L (2017). National assessment of data quality and associated systems-level factors in Malawi. Glob Health Sci Pract.

[19] Rowe AK (2009). Potential of integrated continuous surveys and quality management to support monitoring, evaluation, and the scale-up of health interventions in developing countries. Am J Trop Med Hyg.

[20] Mutale W, Chintu N, Amoroso C, Awoonor-Williams K, Phillips J, Baynes C, Michel C, Taylor A, Sherr K (2013). Improving health information systems for decision making across five sub-Saharan African countries: implementation strategies from the African health initiative. BMC Health Serv Res.

[21] Patnaik S, Brunskill E, Thies W. Evaluating the accuracy of data collection on mobile phones: A study of forms, SMS, and voice. In: 2009 International Conference on Information and Communication Technologies and Development (ICTD). Institute of Electrical and Electronics Engineers (IEEE); 2009. p. 74–84. https://ieeexplore.ieee.org/abstract/document/5426700.

[22] Tomlinson M, Solomon W, Singh Y, Doherty T, Chopra M, Ijumba P (2009). The use of mobile phones as a data collection tool: a report from a household survey in South Africa. BMC Med Inform Decis Mak.

[23] Dabalen A, Etang A, Hoogeveen J, Mushi E, Schipper Y, von Engelhardt J (2016). Mobile phone panel surveys in developing countries: a practical guide for microdata collection.

[24] Pew Research Center (2015). Cell phones in Africa: communication lifeline.

[25] Gibson Dustin G, Pereira Amanda, Farrenkopf Brooke A, Labrique Alain B, Pariyo George W, Hyder Adnan A (2017). Mobile Phone Surveys for Collecting Population-Level Estimates in Low- and Middle-Income Countries: A Literature Review. Journal of Medical Internet Research.

[26] Heidkamp R, NEP Working Group (2017). The national evaluation platform for maternal, newborn, and child health, and nutrition: from idea to implementation. J Glob Health.

[27] Chipokosa S, Pattnaik A, Misomali A, Mohan D, Peters M, Kachale F, Ndawala J, Marx M. How strong are Malawi’s family planning programs for adolescent and adult women? Results of a national assessment of implementation strength conducted by Malawi’s national evaluation platform. J Glob Health. n.d.; In press. 10.7189/jogh.09.020901 PMC7689283 33282227

[28] Corkrey R, Parkinson L (2002). Interactive voice response: review of studies 1989-2000. Behav Res Methods Instrum Comput.

[29] Jackle A, Roberts C, Lynn P (2010). Assessing the effect of data collection mode on measurement. Int Stat Rev.

[30] Groves R (2011). Three eras of survey research. Public Opin Q.

[31] Greenleaf AR (2017). Building the evidence base for remote data collection in low- and middle-income countries: comparing reliability and accuracy across survey modalities. J Med Internet Res.

